# Zinc mercury(II) tetra­kis­(seleno­cyanate)

**DOI:** 10.1107/S1600536813022502

**Published:** 2013-08-17

**Authors:** Hai-Qing Sun, Xin-Qiang Wang, Wei-Wei Zhang

**Affiliations:** a School of Materials Science and Engineering, Shandong University of Science and Technology, Qingdao 266590, People’s Republic of China; b State Key Laboratory of Crystal Materials (Shandong University), Jinan 250100, People’s Republic of China

## Abstract

The title crystal, [HgZn(NCSe)_4_]_*n*_, a coordination polymer, has a diamond-like network. In the crystal, the metal ions, Zn^2+^ and Hg^2+^, are both located on fourfold inversion axes and mimic the role of C atoms in the structure of diamond, and the linear seleno­cyanate bridges replace the C—C bonds. The C—N—Zn unit is almost linear and the C—Se—Hg unit is nearly a right angle. Thus, the HgZn_4_ (or ZnHg_4_) arrangement is midway between a tetra­hedron and a square plane, with two types of Hg—Zn—Hg (or Zn—Hg—Zn) angles of 92.38 (6) and 156.45 (6)°.

## Related literature

For background to coordination polymers, see: Batten *et al.* (2009 ▶). For diamond-like networks, see: Sun *et al.* (2006 ▶); Evans *et al.* (1999 ▶). For similar structures, see: Wang *et al.* (2001 ▶, 2007 ▶); Sun *et al.* (2005 ▶, 2006 ▶); Tian *et al.* (1999 ▶); Xu *et al.* (1999 ▶); Yan *et al.* (1999 ▶); Yuan *et al.* (1997 ▶).

## Experimental

### 

#### Crystal data

[HgZn(NCSe)_4_]
*M* *_r_* = 685.88
Tetragonal, 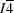

*a* = 11.2716 (1) Å
*c* = 4.6981 (1) Å
*V* = 596.89 (2) Å^3^
*Z* = 2
Mo *K*α radiation
μ = 27.02 mm^−1^
*T* = 293 K
0.13 × 0.12 × 0.10 mm

#### Data collection

Bruker APEXII CCD area-detector diffractometer
Absorption correction: multi-scan (*APEX2*; Bruker, 2005 ▶) *T*_min_ = 0.127, *T*_max_ = 0.173
2476 measured reflections
1244 independent reflections
1113 reflections with *I* > 2σ(*I*)
*R*_int_ = 0.029

#### Refinement

*R*[*F* ^2^ > 2σ(*F* ^2^)] = 0.026
*wR*(*F* ^2^) = 0.053
*S* = 0.86
1244 reflections
33 parameters
Δρ_max_ = 1.35 e Å^−3^
Δρ_min_ = −0.81 e Å^−3^
Absolute structure: Flack (1983 ▶), 467 Friedel pairs
Absolute structure parameter: 0.026 (10)

### 

Data collection: *APEX2* (Bruker, 2005 ▶); cell refinement: *SAINT* (Bruker, 2005 ▶); data reduction: *SAINT*; program(s) used to solve structure: *SIR97* (Altomare *et al.*, 1999 ▶); program(s) used to refine structure: *SHELXL97* (Sheldrick, 2008 ▶); molecular graphics: *SHELXTL* (Sheldrick, 2008 ▶); software used to prepare material for publication: *WinGX* (Farrugia, 2012 ▶).

## Supplementary Material

Crystal structure: contains datablock(s) I, global. DOI: 10.1107/S1600536813022502/br2230sup1.cif

Structure factors: contains datablock(s) I. DOI: 10.1107/S1600536813022502/br2230Isup2.hkl

Additional supplementary materials:  crystallographic information; 3D view; checkCIF report

## Figures and Tables

**Fig. 1.**
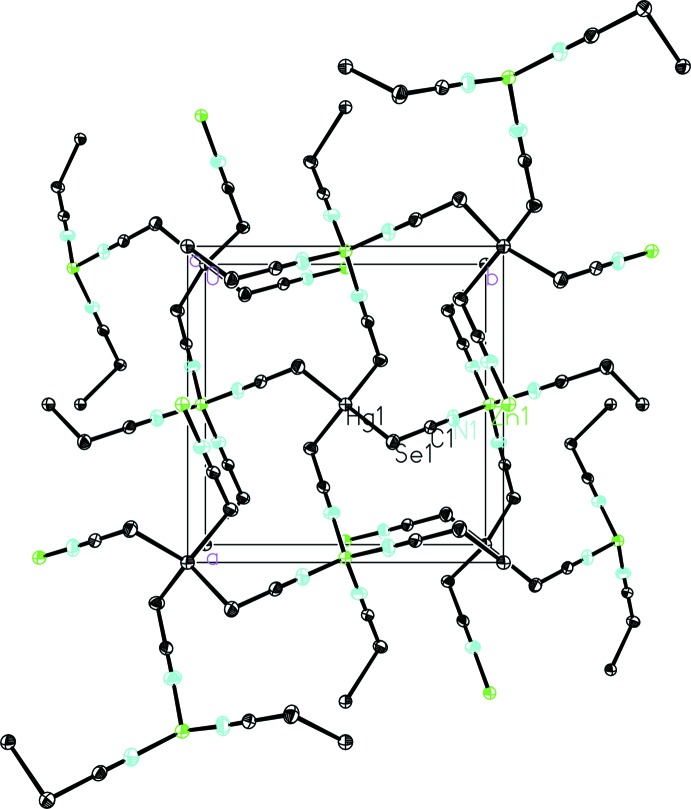
Packing diagram of [ZnHg(NCSe)4]n (viewed down the c axis), with displacement ellipsoids drawn at the 50% probability level.

## supplementary crystallographic information

### 1. Comment

Coordination polymers, which contain ions linked by coordinated ligands into an infinite array, have extensive applications such as porosity, magnetism, non-linear optical activity, reactive networks, heterogenous catalysis and luminescence (Batten *et al.* 2009). [AB(SCN)_4_]_n_ and [AB(SeCN)_4_]_n_ (where A and B = Zn, Cd, Hg or Mn) are coordination polymers that have non-linear optical property (Wang *et al.*, 2007, 2001; Sun *et al.*, 2006, 2005; Yuan *et al.* 1997). [ZnHg(SeCN)_4_]_n_ is a new member of this group.

All the [AB(SCN)_4_]_n_ and [AB(SeCN)_4_]_n_ have similar structure. The [ZnHg(SeCN)_4_]_n_ is of no exception. The Zn and Hg atoms are connected by SeCN^-^ ions, forming an infinite three-dimensional network (see Fig. 1). Each Zn or Hg node is 4-coordinated with Zn—N or Hg—Se bond. The ZnN_4_ and HgSe_4_ tetrahedra are slightly distorted from an ideal one. The Zn—N—C—Se is nearly linear, but the C—Se—Hg is bent. The whole structure might be defined as a diamond-like network with Zn and Hg nodes and bent bonds. The HgZn_4_ (or ZnHg_4_) tetrahedra have a significant distortion from an ideal one, with two types of Hg—Zn—Hg (or Zn—Hg—Zn) angles, 92.38 (6)° and 156.45 (6)°. Along the c-direction there are irregular octagon channels.

### 2. Experimental

Sodium metasilicate nonahydrate (Na_2_SiO_3_.9H_2_O), ZnCl_2_ and KSeCN solution were mixed together with stirring for 1 h. Then the sol is put into a test tube. Glacial acetic acid was added to adjust pH to 3.1. The above solution was sealed and gelled on standing for 72 h. Then some HgCl_2_ solution was added on top of the gel. Within 20 d the ZMSC crystal grew in the gel medium.

### 3. Refinement

The unusually large residual electron density (1.347 e Å^-3^) is found near the Hg atoms.

### Crystal data

**Table:**

[HgZn(NCSe)_4_]	*D*_x_ = 3.816 Mg m^−^^3^
*M**_r_* = 685.88	Mo *K*α radiation, λ = 0.71073 Å
Tetragonal, *I*4	Cell parameters from 1556 reflections
Hall symbol: I -4	θ = 2.6–34.8°
*a* = 11.2716 (1) Å	µ = 27.02 mm^−^^1^
*c* = 4.6981 (1) Å	*T* = 293 K
*V* = 596.89 (2) Å^3^	Prism, colourless
*Z* = 2	0.13 × 0.12 × 0.10 mm
*F*(000) = 596	

### Data collection

**Table:**

Bruker APEXII CCD area-detector diffractometer	1244 independent reflections
Radiation source: fine-focus sealed tube	1113 reflections with *I* > 2σ(*I*)
Graphite monochromator	*R*_int_ = 0.029
φ and ω scans	θ_max_ = 38.1°, θ_min_ = 2.6°
Absorption correction: multi-scan (*APEX2*; Bruker, 2005)	*h* = −16→19
*T*_min_ = 0.127, *T*_max_ = 0.173	*k* = −17→11
2476 measured reflections	*l* = −6→7

### Refinement

**Table:**

Refinement on *F*^2^	Secondary atom site location: difference Fourier map
Least-squares matrix: full	*w* = 1/[σ^2^(*F*_o_^2^)]
*R*[*F*^2^ > 2σ(*F*^2^)] = 0.026	(Δ/σ)_max_ < 0.001
*wR*(*F*^2^) = 0.053	Δρ_max_ = 1.35 e Å^−^^3^
*S* = 0.86	Δρ_min_ = −0.81 e Å^−^^3^
1244 reflections	Extinction correction: *SHELXL97* (Sheldrick, 2008), Fc^*^=kFc[1+0.001xFc^2^λ^3^/sin(2θ)]^-1/4^
33 parameters	Extinction coefficient: 0.0047 (3)
0 restraints	Absolute structure: Flack (1983), 467 Friedel pairs
Primary atom site location: isomorphous structure methods	Absolute structure parameter: 0.026 (10)

### Special details

**Table:**

Geometry. All e.s.d.'s (except the e.s.d. in the dihedral angle between two l.s. planes) are estimated using the full covariance matrix. The cell e.s.d.'s are taken into account individually in the estimation of e.s.d.'s in distances, angles and torsion angles; correlations between e.s.d.'s in cell parameters are only used when they are defined by crystal symmetry. An approximate (isotropic) treatment of cell e.s.d.'s is used for estimating e.s.d.'s involving l.s. planes.
Refinement. Refinement of *F*^2^ against ALL reflections. The weighted *R*-factor *wR* and goodness of fit *S* are based on *F*^2^, conventional *R*-factors *R* are based on *F*, with *F* set to zero for negative *F*^2^. The threshold expression of *F*^2^ > σ(*F*^2^) is used only for calculating *R*-factors(gt) *etc*. and is not relevant to the choice of reflections for refinement. *R*-factors based on *F*^2^ are statistically about twice as large as those based on *F*, and *R*-factors based on ALL data will be even larger.

### Fractional atomic coordinates and isotropic or equivalent isotropic displacement parameters (Å^2^)

**Table:**

	*x*	*y*	*z*	*U*_iso_*/*U*_eq_	
Hg1	0.0000	0.0000	0.0000	0.02909 (11)	
Se1	0.12160 (4)	0.15390 (4)	0.31572 (11)	0.03115 (13)	
Zn1	0.0000	0.5000	−0.2500	0.0289 (2)	
N1	0.0462 (4)	0.3643 (3)	−0.0118 (16)	0.0374 (9)	
C1	0.0756 (4)	0.2818 (4)	0.1106 (10)	0.0280 (9)	

### Atomic displacement parameters (Å^2^)

**Table:**

	*U*^11^	*U*^22^	*U*^33^	*U*^12^	*U*^13^	*U*^23^
Hg1	0.02405 (11)	0.02405 (11)	0.0392 (2)	0.000	0.000	0.000
Se1	0.0322 (2)	0.0250 (2)	0.0363 (3)	−0.00208 (18)	−0.0073 (2)	0.00031 (19)
Zn1	0.0242 (3)	0.0242 (3)	0.0382 (6)	0.000	0.000	0.000
N1	0.040 (2)	0.0242 (16)	0.048 (2)	0.0011 (14)	−0.004 (3)	−0.004 (2)
C1	0.0241 (19)	0.0219 (18)	0.038 (2)	−0.0019 (15)	0.0023 (17)	−0.0046 (17)

### Geometric parameters (Å, º)

**Table:**

Hg1—Se1	2.6623 (5)	Zn1—N1^iv^	1.966 (6)
Hg1—Se1^i^	2.6623 (5)	Zn1—N1^v^	1.966 (6)
Hg1—Se1^ii^	2.6623 (5)	Zn1—N1^vi^	1.966 (6)
Hg1—Se1^iii^	2.6623 (5)	Zn1—N1	1.966 (6)
Se1—C1	1.810 (5)	N1—C1	1.141 (7)
			
Se1—Hg1—Se1^i^	108.084 (11)	N1^iv^—Zn1—N1^vi^	110.6 (4)
Se1—Hg1—Se1^ii^	112.28 (2)	N1^v^—Zn1—N1^vi^	108.91 (18)
Se1^i^—Hg1—Se1^ii^	108.084 (11)	N1^iv^—Zn1—N1	108.91 (18)
Se1—Hg1—Se1^iii^	108.084 (11)	N1^v^—Zn1—N1	110.6 (4)
Se1^i^—Hg1—Se1^iii^	112.28 (2)	N1^vi^—Zn1—N1	108.91 (18)
Se1^ii^—Hg1—Se1^iii^	108.084 (11)	C1—N1—Zn1	175.5 (6)
C1—Se1—Hg1	94.29 (14)	N1—C1—Se1	178.1 (5)
N1^iv^—Zn1—N1^v^	108.91 (18)		
			
Se1^i^—Hg1—Se1—C1	−14.76 (14)	N1^iv^—Zn1—N1—C1	69 (5)
Se1^ii^—Hg1—Se1—C1	−133.89 (14)	N1^vi^—Zn1—N1—C1	−52 (5)
Se1^iii^—Hg1—Se1—C1	106.99 (14)	Hg1—Se1—C1—N1	140 (12)

Symmetry codes: (i) −*y*, *x*, −*z*; (ii) −*x*, −*y*, *z*; (iii) *y*, −*x*, −*z*; (iv) *y*−1/2, −*x*+1/2, −*z*−1/2; (v) −*x*, −*y*+1, *z*; (vi) −*y*+1/2, *x*+1/2, −*z*−1/2.
